# Magneto-thermochromic coupling Janus sphere for dual response display†

**DOI:** 10.1039/c9ra02892g

**Published:** 2019-06-07

**Authors:** Yiwen Cui, Yu Wang, Jie Wu, Xiaokang He, Shouhu Xuan, Xinglong Gong

**Affiliations:** CAS Key Laboratory of Mechanical Behavior and Design of Materials, Department of Modern Mechanics, CAS Center for Excellence in Complex System Mechanics, University of Science and Technology of China Hefei Anhui 230027 China gongxl@ustc.edu.cn xuansh@ustc.edu.cn wyu@ustc.edu.cn +86 551 63600419 +8655163601702 +8655163601236

## Abstract

This work demonstrates a simple microfluidic device to synthesize a magneto-thermochromic sphere with Janus inner structure. The Janus sphere is composed of Fe_3_O_4_ microspheres, thermochromic particles, and polyacrylamide matrix. Because the Fe_3_O_4_ microspheres are assembled together in one pole, the Janus sphere can turn around by varying the direction of the external magnetic field. Originating from the temperature-dependent property of the thermochromic particles, the final Janus sphere can change its color from red to pale blue when the temperature is increased from 5 to 45 °C. The detailed formation process and the magneto-thermochromic mechanism are carefully investigated. Due to the magnetic switch and thermochromism, these Janus spheres can be applied as colorful displays by controlling the magnetic field and temperature. The results demonstrate that the dual responsive Janus spheres possess broad application potential in temperature sensors and displays.

## Abstract 2

This work demonstrates a simple microfluidic device to synthesize a magneto-thermochromic sphere with Janus inner structure.

## Graphical abstract

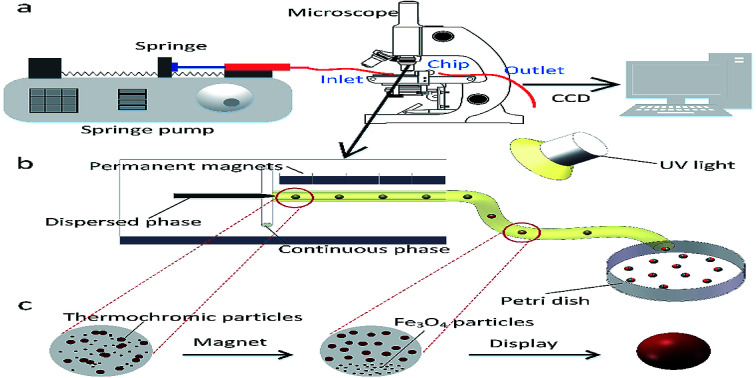

## Introduction

Janus spheres (JPs) are made up of two or more different materials, and the surface of JPs can even produce two distinct types of chemical reactions on a single sphere.^1^ Due to their biphasic characteristic, JPs have received increasing attention in remote manipulation,^2,3^ drug delivery,^4^ switchable display devices,^5–10^ imaging, and magnetoelastic therapy.^11^ Many approaches have been developed to fabricate JPs, including masking,^12^ self-assembly,^13^ phase separation,^14^ physical vapor deposition^15^ and microfluidics.^16–18^ Microfluidics is the science and technology of a system that process or manipulate small amounts of fluidics.^19^ With the development of microfluidic technology, microfluidics has gradually become the principal method to fabricate JPs because of the following advantages: the ability to precisely adjust sphere size, control sphere shape, low sample consumption, and easy production of composite spheres.^20–23^

There are two methods for producing Janus droplets by microfluidic technology. UV irradiation method is used to prepare JPs from Janus droplets containing a photoinitiator.^24–27^ Photopolymerization is performed immediately, as soon as the droplet is formed. The curing time can be extended indefinitely as long as the two segments are immiscible. Currently, this method is the most widely used one for solidification.^24–26^ However, many reports based on UV lights often increase the length of outlet channel to prolong the reaction time,^28,29^ which cause the spheres to be easily clogged in the channel. Phase separation is another method to create JPs in single emulsion droplet. For this method, the W/O emulsions are prepared by photopolymer UV-mediated phase separation of the polymer JPs.^30^ In comparison to UV irradiation, this method is more complicated. During the past decades, various JPs have been prepared by the microfluidic. For example, different shapes of JPs (spherical, cylindrical, disc-shaped, dumbbell-shaped),^31^ organic and inorganic composites of JPs,^32^ JPs composed of different polysaccharides,^33^ and biodegradable JPs doped with drug molecules.^34^ In consideration of the wide application, more efforts should be conducted to develop high efficient method to synthesize multifunctional JPs.

JPs with color anisotropy is a special concern owing to their excellent optical performance.^35,36^ Among them, magnetic JPs^37^ created from magnetic materials has attracted intensive interests because of their dual properties, such as magnetic switch and black color. Guided by an external magnetic field^38^ and the induced spatial manipulation of the polydisperse magnetic JPs,^39^ these multifunctional JPs can be applied in optical display.^40,41^ Previously, Luo *et al.*^42^ reported the photonic crystal balls and the structural colors of the balls could be regulated by both magnetic field and temperature under UV irradiation. Simultaneously, the magneto-thermochromic Janus spheres which can sense the environment temperature also have been fabricated and they shed a new sight on display field.^43^ Although the combination of thermochromism and magnetism is attractive in colorful display due to its changeable color characteristics, easy preparation and wide temperature (especially low temperature) responsibility are the two challenges for the magneto-thermochromic Janus spheres, and thus confined their practical application. Moreover, the detailed elucidation of the magneto-thermochromic coupling mechanism is also required.

Here, we demonstrated a simple microfluidic device to synthesize Janus spheres which could be both sensitive to temperature and magnetic field. A simple two-phase microfluidic technology, UV lights and magnets were set up to construct JPs by combining Fe_3_O_4_ microspheres, thermochromic particles and polyacrylamide (PAM) as building blocks. The color of the JPs changed in a wide low temperature range (5–45 °C) and the position switch of the JPs could be realized with a magnetic pen. A display screen composed by temperature-magnetism dual response JPs was developed and the controlling strategy was discussed. These JPs would spontaneously aggregate by turning on the previously constructed magnetic field. The combined application in display and temperature sensor derived from the magneto-thermochromic JPs will facilitate the progress in the field of exhibition equips and sensors.

## Experimental

### Design and fabrication of the microfluidic platform

The polydimethylsiloxane (PDMS) microfluidic platform was fabricated by standard soft lithography. First, a layout editor (CorelDRAW) was printed on a film as the photomask in the photolithography process. Next, the negative SU-8 photoresist (SU-8-2075, MicroChem) was coated on a Si wafer, then placed on a spin coater to fabricate molds. The spin coating process was set at 1000 rpm for 18 s and 3000 rpm for 1 min. The thickness of the obtained photoresist was 70–100 μm. Then, the prepared dry photomask film was placed on top of the photoresist. It was illuminated for 2 min by the UV lamp (M365L2-C1, Thorlabs). Developer, ethanol and deionized water were employed to wash the Si wafer repeatedly until the non-transparent part of the glue was totally washed away. Then, the master mold was obtained.

Next, PDMS mixture (curing agent ratio: 10 : 1) was poured onto the master mold. After degassing in a vacuum desiccator for 5 min, it was cured in a convection oven at 90 °C for 20 min. Then, the solidified PDMS was peeled and punched by a manual puncher (Harris Uni-Core, World Precision Instruments). The inlet with a diameter of 1.2 mm was fabricated. The cast PDMS was rinsed by ethanol for 5 min, and then washed by distilled water. After the cleaning process, the PDMS parts were placed in an oven at 80 °C for 5 min to dry the surface. Then, the grooved PDMS and another flat PDMS were bonded by oxygen plasma treatment (WU-1000, Wenhao). Finally, an initial chip was obtained.

The above chip was cut at the point which was 5 mm from the throat in the outlet channel. Then, a polyethylene tube (PE-10, Smiths Medical, 0.28 mm ID, 0.61 mm OD) was inserted into the chip as the new outlet channel. There were two inlets connecting two polyethylene tubes (Smith medical, 0.38 mm ID, 1.09 mm OD). Before using, the microfluidic chip was put into an oven at 65 °C for 24 h to ensure the hydrophobicity. Finally, a microfluidic device for fabricating the JPs was achieved.

### Synthesis of Fe_3_O_4_ microspheres

Fe_3_O_4_ microspheres were prepared by a solvothermal method. Briefly, FeCl_3_·6H_2_O (1.08 g), NaAc (4.0 g) and polyacrylic acid (PAA) (100 mg) were dissolved in ethylene glycol (EG) (10 mL) and diethylene glycol (DEG) (30 mL) in a beaker (100 mL) by magnetic stirring. 30 min later, the obtained homogeneous yellow solution was transferred to a Teflon-lined stainless steel autoclave (100 mL) and heated at 200 °C for 12 h. Then, the autoclave was cooled down to room temperature (25 °C) and the obtained magnetic microspheres were washed with ethanol and deionized water, respectively. Finally, these microspheres were dispersed in deionized water with 8.7% mass concentration, and the ferrofluid with a mass fraction of 8.7% was obtained.

### Preparation of polyacrylamide (PAM) spheres

The PAM spheres were synthesized *via* photopolymerization. Briefly, the PAM precursor solution (deionized water (8.7 mL), 2-hydroxyl-2-methyl-1-phenyl-1-propanone (HMPP, 0.1 mL), acrylamide (AM, 0.661 g), *N*,*N*′-methylene-bisacrylamide (0.4 g), sodium dodecyl sulfate (SDS, 25 μL) and Tween 25 (25 μL)) were added into a 100 mL flask equipped with a magnetic stirrer. Among them, SDS was added as a surfactant to prevent droplet fusion and HMPP was added as the photoinitiator to induce the conversion of droplets to particles.

After being fully mixed evenly under a dark environment, the obtained solution was used as the dispersed phase, and mineral oil was used as the continuous phase. They were introduced into the injector of 1 mL, respectively. In particular, the polythene tube of dispersed phase needed to avoid light, so they were wrapped with aluminum foil to prevent irradiating. Then, the equipped syringe pumps (LSP02-1B, Longer-Pump) were used to promote the liquid. During the preparation, four UV lights (wavelength is from 280 nm to 320 nm; the peak is 308 nm; power is 6 W) were used to initiate the droplet solidification chemical reaction. A high-speed video camera (Phantom v2512, Vision Research Inc.) was used to capture the droplet formation process.

### Synthesis of thermochromic polyacrylamide (TPAM) spheres

Similar to the PAM spheres, the synthesis of thermochromic polyacrylamide (TPAM) spheres were prepared through UV-irradiation in the downstream of the chip. 0.514 g of thermochromic particles (Shen Zhen Color Change Chemical Technology Co, Ltd.) and 9.7571 g of PAM mixture solution were added in a 15 mL beaker and the solution was collected in a shaded bottle as the dispersed phase. When the dispersed phase and the continuous phase passed into the microfluidic chip, a series of stable droplets was formed. Then, the droplets were converted to TPAM spheres through the photosynthetic method. There are two kinds of thermochromic particles. One was red particles (RTP) with its color change in the range of 5–25 °C. The other was the blue particles (BTP), whose color would change in the range of 25–45 °C. Through mixing RTP and BTP (mass ratio = 2 : 1, MTP), the mixture could sense the temperature change between 5 °C and 45 °C. The detailed composition of thermochromic particles is shown in Tables 1 and 2 (ESI).† The color of the thermochromic particles is dependent on the molecular structure. Since the molecular structure of thermochromic particle is varied at different temperature, the color changes by turning the temperature.

### Preparation of magnetic polyacrylamide (MPAM) spheres

The MPAM spheres were prepared with the introduction of Fe_3_O_4_ microspheres. The ferrofluid with a mass fraction of 8.7% (0.1 mL) and PAM mixture solution (0.2 mL) were mixed and the as-obtained solution was collected in a shaded injector as the dispersed phase. The mineral oil was used as the continuous phase. Then, the MPAM droplets were formed *via* a microfluidic device. MPAM spheres were converted by droplets through the irradiation of ultraviolet lamps.

### Preparation of magneto-thermochromic polyacrylamide (MTPAM) dual response JPs

Here, the permanent neodymium iron boron (NdFeB) magnets were located on the microfluidic channel system to supply the magnetic field. 0.2 mL TPAM solution (5 wt%), 0.1 mL Fe_3_O_4_ solution (8.7 wt%) were homogeneously mixed as the dispersed phase before preparation. The mineral oil phase was used as the continuous phase. Then, the MTPAM droplets were formed *via* a microfluidic device and they were converted to the MTPAM JPs through the irradiation of ultraviolet lamps.

## Results and discussion

**Fig. 1:**
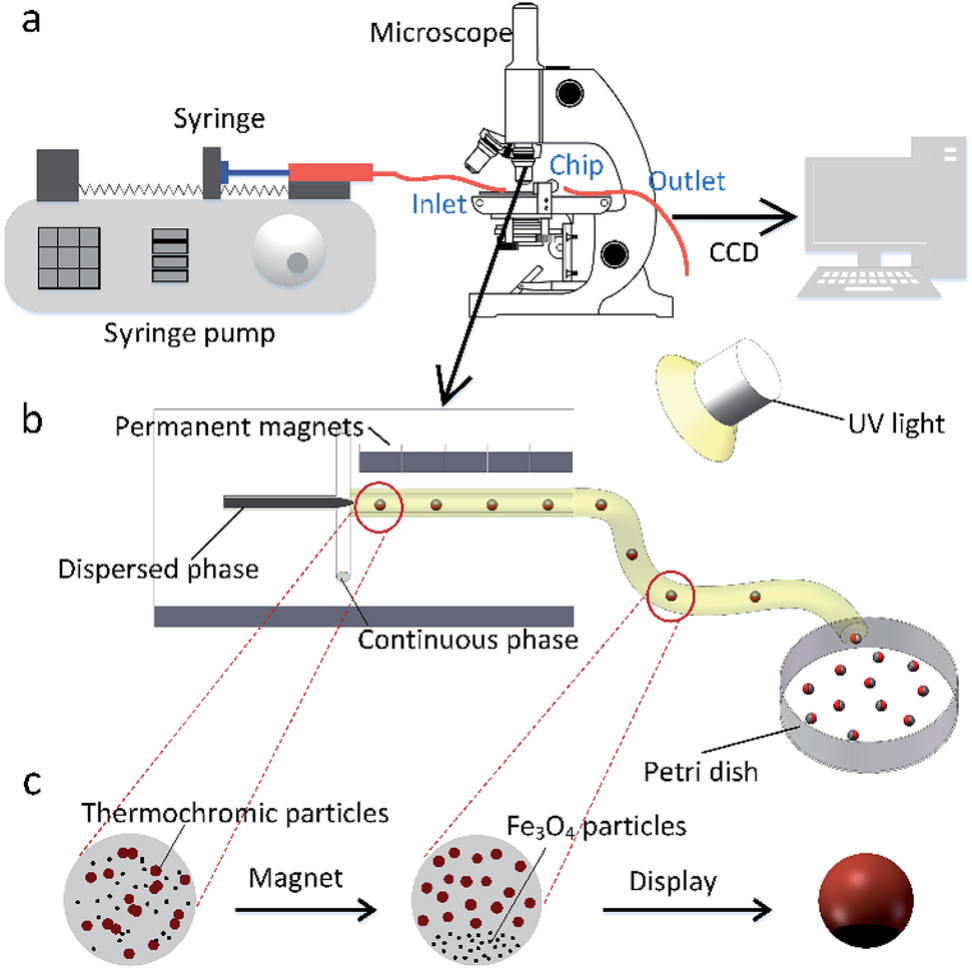
(a) Schematic diagram of the experimental setup. Two high-precision syringe pumps pushed the dispersed phase and the continuous phase into the chip. (b) Schematic diagram of the microfluidic device. (c) Structure change diagram of a Janus droplet.

In this work, the microfluidic device is firstly constructed and it can be used for preparing various spheres, such as the polyacrylamide (PAM), thermochromic polyacrylamide (TPAM), magnetic polyacrylamide (MPAM), and magneto-thermochromic polyacrylamide (MTPAM) JPs. During the synthesis process, the PAM precursor solution is set as the dispersed phase and mineral oil is used as the continuous phase. Then, the droplets are formed by utilizing a microfluidic flow-focusing device (Fig. 1a). After further crosslinking under the UV irradiation, the following spheres are obtained. The TPAM and MPAM spheres are prepared by mixing the functional materials (Fe_3_O_4_ microspheres and thermochromic particles) with the PAM precursor solution as the dispersed phase. For MTPAM JPs, a column of permanent magnets are fixed nearby the microfluidic channel and the magnetic field density is controlled by varying the distance (Fig. 1b). Under the action of magnet field, the Fe_3_O_4_ microspheres are assembled together in one pole of droplet to form a Janus structure (Fig. 1c).

**Fig. 2:**
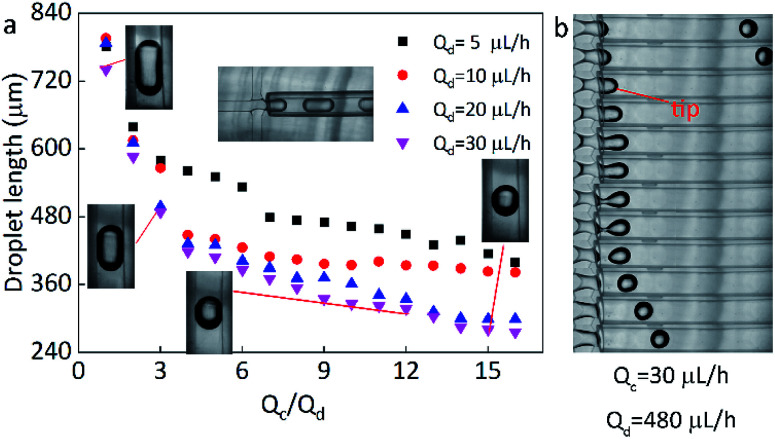
(a) The image of the relationship between droplet length and *Q*_c_/*Q*_d_. The dots of black, red, blue and pink represent the *Q*_d_ of 5, 10, 20, 30 μL h^−1^, respectively. (b) The picture of a droplet forming process under *Q*_c_ = 480 μL h^−1^, *Q*_d_ = 30 μL h^−1^, the length of the droplet is about 250 μm. The time interval between each picture is 0.1 s.

Firstly, the parameters for the generated droplets are investigated. The shape and length of droplets are dependent on the flow rate of the continuous phase (*Q*_c_) and the dispersed phase (*Q*_d_) (Fig. 2a). When *Q*_c_ is small, a thin jet is formed and elongated downstream far from the cross-junction. At the tip of the jet, the droplet started to grow up until the jet no longer drag the droplet, then the droplet was separated and moved away, and another droplet started to grow (Fig. 2b). As *Q*_c_/*Q*_d_ increased under a fixed *Q*_d_, the pressure of the continuous phase increased. The pressure of continuous phase tended to cut the dispersed phase and the next formed droplet was shorter than the previous one. Finally, the shape of the droplet changed from a long ellipsoid to a sphere. When *Q*_d_ = 30 μL h^−1^, *Q*_c_ = 30 μL h^−1^, the shape of droplets was a long ellipsoid. With the increasing of *Q*_c_, the length of the droplets decreased and toward to the spheres. When *Q*_c_ = 480 μL h^−1^, a spherical droplet with average diameter of 250 μm was achieved. In addition, when *Q*_c_/*Q*_d_ was fixed, the diameters of droplets decreased with the increasing of *Q*_d_. For example, the length of droplets decreased from 570 μm to 490 μm approximately as *Q*_d_ increased from 5 μL h^−1^ to 30 μL h^−1^ under *Q*_c_/*Q*_d_ = 3. Therefore, the shape and size of the droplets can be varied by controlling the *Q*_c_, *Q*_d_, and their ratio.

**Fig. 3:**
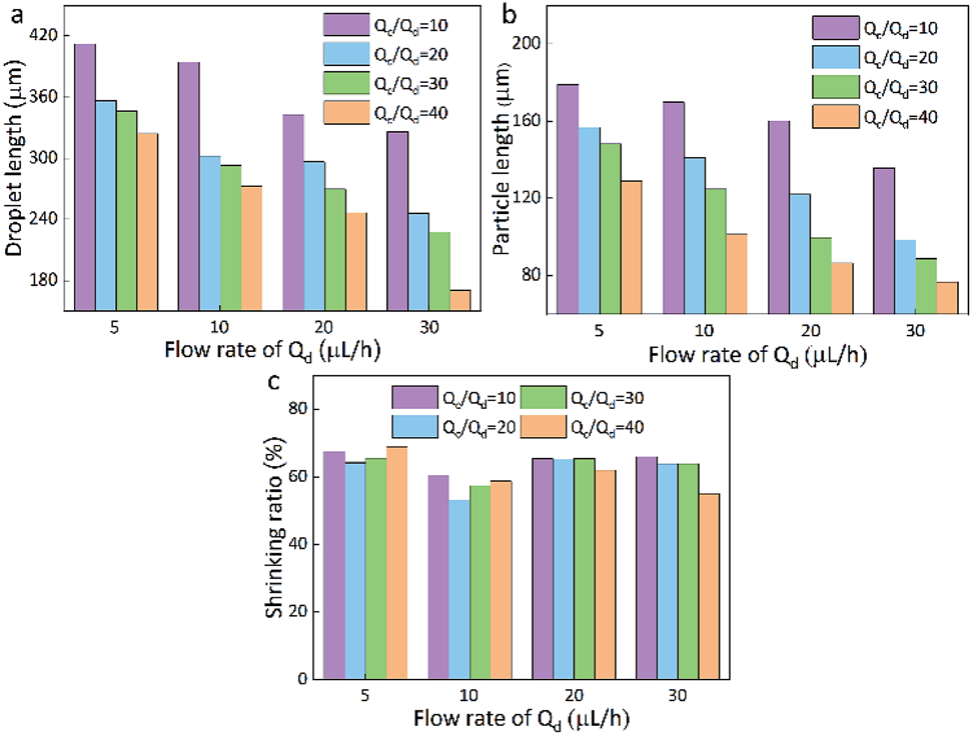
Droplet length (a), particles length (b), and the shrinking ratio (c) of PAM in different continuous/dispersed (*Q*_c_/*Q*_d_) flow rates.

Here, the size of the PAM precursor droplets often reduces after the cross-liking by illumination of UV lights. The size relationship between the droplets and spheres were studied to search for optimal *Q*_c_ and *Q*_d_. The sizes of droplets and spheres were calculated by analyzing the picture (Leica DM500) on the computer. Keep the *Q*_d_ as a constant, the length of droplet significantly decreased with the increase of *Q*_c_/*Q*_d_. When *Q*_c_/*Q*_d_ was fixed, the droplet length decreased as *Q*_d_ increased. Similarly, the length of these spheres exhibited the same tendency as the droplets length. The particle length decreased with the increasing of *Q*_c_/*Q*_d_ at a fixed *Q*_d_. In addition, the length of particles decreased as *Q*_d_ increased when *Q*_c_/*Q*_d_ was fixed. For example, the length of particles decreased from 130 μm to 70 μm approximately as *Q*_d_ increased from 5 μL h^−1^ to 30 μL h^−1^ under *Q*_c_/*Q*_d_ = 40. And the particle length decreased from 140 μm to 70 μm with the increasing of *Q*_c_/*Q*_d_ at a fixed *Q*_d_ = 30 μL h^−1^(Fig. 3).

In comparison to the droplets, the size of the spheres is smaller. During the transformation, the irradiation of UV lamps is conducted and the water in the droplets is evaporated and the PAM networks shrink during the crosslinking process, thus the size of the droplets is reduced after the solidification. By calculation, it is found that the spheres have an approximately stable shrinkage ratio of 60%. Based on the experiments, it was found that when *Q*_c_ = 200 μL h^−1^, *Q*_d_ = 20 μL h^−1^, spherical particles could be produced quickly and easily. Therefore, this condition is kept as a constant for the following preparation of multifunctional spheres.

**Fig. 4:**
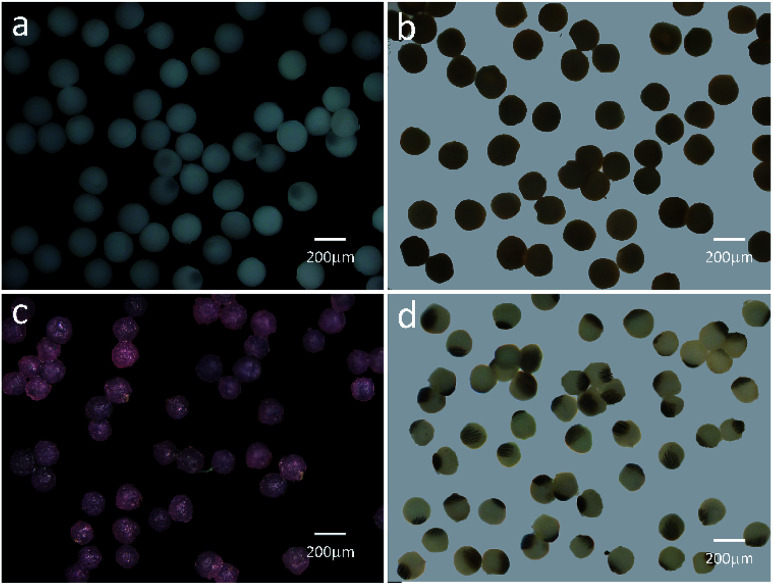
(a and b) Optical micrographs of PAM, MPAM spheres. (c) Optical micrograph of TPAM spheres at 15 °C. (d) Optical microscopy image of MTPAM JPs with RTP at room temperature (25 °C). The scale bar is 200 μm.

Based on the above result, the PAM, MPAM, and TPAM spheres are prepared by the optimum flow rate of *Q*_c_ = 200 μL h^−1^ and *Q*_d_ = 20 μL h^−1^. As shown in Fig. 4a, the optical micrographs of the PAM spheres exhibit a uniform shape with an average diameter of about 160 μm. Different from the white colored PAM spheres, the MPAM spheres are pale brown, which must be responded for the presence of black Fe_3_O_4_ microspheres. The Fe_3_O_4_ microspheres are uniformly dispersed within the MPAM spheres because the magnetic field has not been applied here (Fig. 4b). In this work, the average size of the MPAM is similar to the PAM spheres. By using the same method, the TPAM spheres with temperature dependent color can be also achieved by introducing the thermochromic materials. As shown in Fig. 4c, the TPAM spheres with 160 μm are well dispersed and they show red color due to the presence of the thermochromic particles. Different from the above process, a NdFeB magnet array (20 × 10 × 4 mm, 130 mT) should be fixed nearby the outlet channel to fabricate MTPAM spheres (Fig. 1b). Keeping other parameters as constant, the black disperse phase is composed of TPAM precursor, Fe_3_O_4_ microspheres, thermochromic particles and photoinitiators. After the polymerization, the droplets transform to the magneto-thermochromic polyacrylamide (MTPAM) spheres. Clearly, the spheres show a typical Janus structure and it must be originated from the magnetic field (Fig. 4d). The JPs with thermochromic particles exhibit white color at room temperature, while the Fe_3_O_4_ hemispheres present black color, thus the JPs exhibit two distinct colors boundary.

**Fig. 5:**
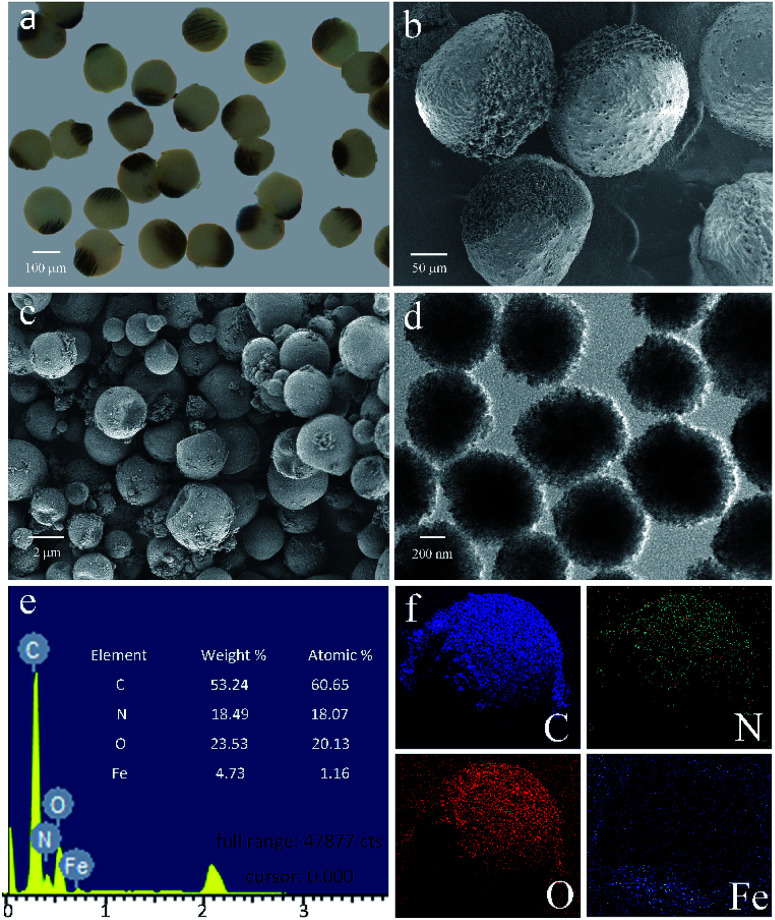
(a) Optical micrograph of MTPAM JPs composed of MTP under room temperature. (b and c) Scanning electron microscope (SEM) images of MTPAM JPs and thermochromic particles. (d) Image of transmission electron microscopy (TEM) of Fe_3_O_4_ microspheres. (e) EDX diagram and its elemental composition. (f) The elemental mapping images.

As shown in the Fig. 5a, besides being concentrated to form the Janus structure, the Fe_3_O_4_ microspheres are also aggregated along the magnetic field and many chains-like structure is clearly observed in the JPs. The magnetic field applying to the channel is tunable by changing the perpendicular distance between the magnet and outlet tube. During the preparation, the Fe_3_O_4_ microspheres in the droplets are attracted and aggregated to one pole. At the same time, the thermochromic materials are squeezed within the whole spheres to form a colored hemisphere. Therefore, after applying the ultraviolet lights on the Janus droplets, magneto-thermochromic polyacrylamide (MTPAM) JPs are successfully prepared by photopolymerization. Fig. 5b shows the typical SEM image of the MTPAM JPs, which also demonstrates the average size of the particles is about 170 μm, agreed with the optical image. Here, a clear boundary is found on the surface of sphere, indicating the Janus structure. In this work, the size of the thermochromic particles and Fe_3_O_4_ microspheres are 2 μm and 300 nm respectively (Fig. 5(c and d)), and they are much smaller than the JPs. To this end, they are difficult to be distinguished in the optical image. However, the chains-like structure aggregated by Fe_3_O_4_ microspheres is observed due to large size. The EDX and mapping images shows the detailed distribution of the Fe, C, N and O elements in the MTPAM JPs (Fig. 5(e and f)). Obviously, the Fe_3_O_4_ microspheres are assembled within one pole of the droplet and forms the Janus structure. Based on the above analysis, it can be found that both the Fe_3_O_4_ microspheres and thermochromic materials are integrated within one Janus sphere, which thus endows them with wonderful magnetic functionality and temperature-dependent color.

**Fig. 6:**
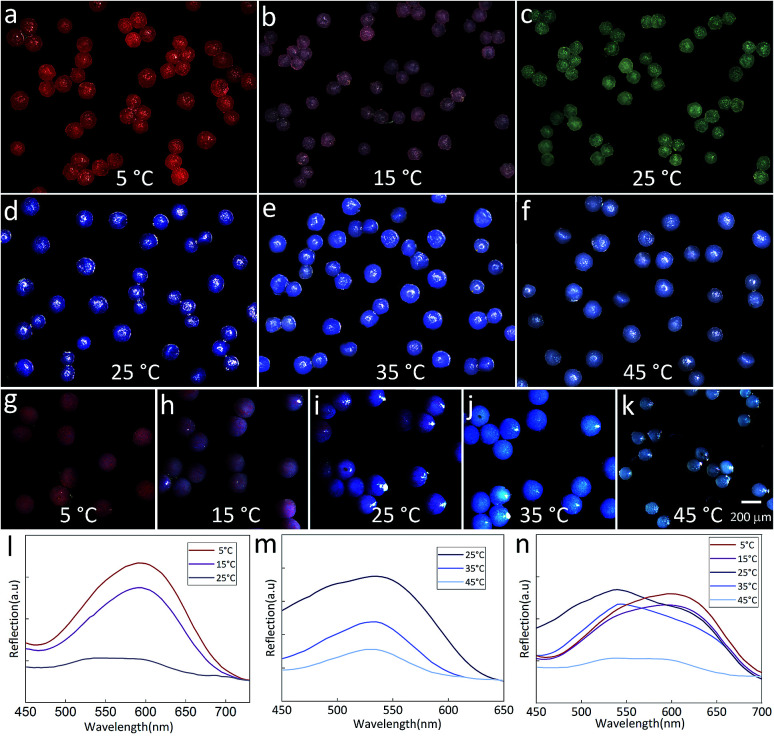
(a–c) The color of TPAM spheres composed of RTP changes with temperature (5–25 °C). (d–f) The images of TPAM spheres composed of BTP changes with temperature (25–45 °C). (g–k) Optical images of TPAM spheres composed of MTP with temperature varied from 5 °C to 45 °C. (l–n) The reflection spectra of TPAM spheres composed of RTP, BTP, MTP, respectively.

The temperature-dependent color of the spheres is originated from the thermochromic materials. Here, two kinds of thermochromic particles are used and their temperature sensitive ranges are 5–25 °C (RTP) and 25–45 °C (BTP). After incorporating these materials into the droplets, the color of the final spheres changes with the external temperature. Fig. 6(a–c) showed the color change of TPAM spheres prepared by RTP. When the temperature was 5 °C, the TPAM showed a deep red color (Fig. 6a). As the temperature rose to 15 °C, the color of these spheres turned reddish purple (Fig. 6b). If the temperature was near 25 °C, the color of the spheres would fade to white (Fig. 6c). Furthermore, this process was reversible, which proved the reliability of these TPAM spheres as a temperature sensor. Similarly, the TPAM spheres composed of BTP showed another color change behavior. With the temperature increased from 25 °C to 35 °C and 45 °C (Fig. 6(d–f)), the color of these spheres varied from dark blue to light blue and pale blue, respectively.

In order to enlarge the temperature sensitive range, a mixture of BTP and RTP was used in fabricating the colorful spheres. Fig. 6(g–k) exhibited the color change of the TPAM prepared by the MTP. When the temperature was 5 °C, the color of these spheres changed from the original bright red to the dull red due to the presence of BTP (Fig. 6g). When the temperature was around 15 °C, these spheres showed a reddish purple color (Fig. 6h). At room temperature (25 °C), the color of the third TPAM spheres was dominated by BTP and showed dark blue color (Fig. 6i). As the temperature continued increased to 35 °C and 45 °C, the spheres turned light blue color (Fig. 6j) and pale white (Fig. 6k).

Fig. 6l showed the reflex spectra of thermochromic polyacrylamide (TPAM) spheres composed of RTP. When the temperature was 5 °C and 15 °C, the colors of these spheres were dark red and pink, and the peak positions of the spectra were about 600 nm. The intensity of peak dropped when temperature increased from 5 °C to 15 °C. As the temperature rose to 25 °C, the color of these spheres changed to white and no obvious peak was found in the spectrum. Fig. 6m showed the spectra of TPAM spheres composed of BTP. With the temperature increased from 25 °C to 35 °C and 45 °C, the peak position was about 540 nm and the intensity of peak was gradually reduced, which indicated the color of spheres was gradually faded. Fig. 6n exhibited the spectra of TPAM composed of MTP. At 5 °C and 15 °C, the peak position was 600 nm, and the intensity of peak reduced with temperature. When the temperature increased to 25 °C and 35 °C, the color of spheres changed into blue and the peak position shifted to 540 nm. With the temperature further increased to 45 °C, the intensity decreased so much that the peak position could be hardly observed. These results were well consistent with the displaying color.

**Fig. 7:**
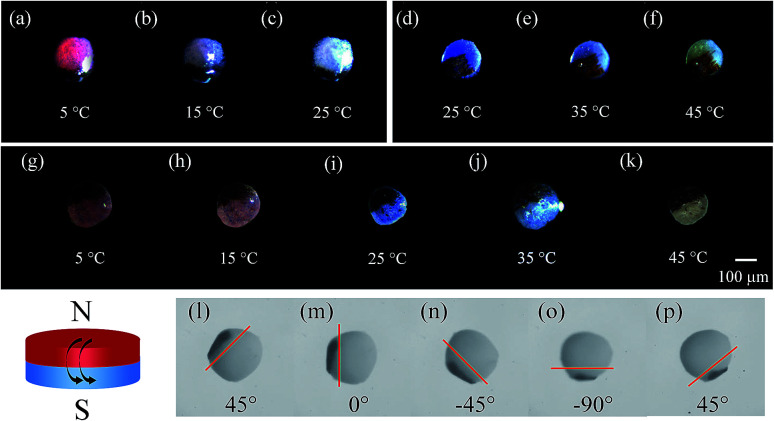
(a–c) The color of MTPAM JPs with RTP changes with temperature (5–25 °C). (d–f) The images of MTPAM JPs with BTP changes with temperature (25–45 °C). (g–k) Optical images of MTPAM JPs with RTP/BTP under different temperature (5–45 °C). (l–p) Schematic illustration and optical images exhibit the rotational motion of a single Janus sphere under an external magnetic field induced by a rotating magnet.

Subsequently, the magneto-thermochromic polyacrylamide (MTPAM) JPs with different temperature sensitivities in the range of 5–25 °C, 25–45 °C, and 5–45 °C are achieved by using the microfluidic device. As shown in Fig. 7(a–k), the color of MTPAM JPs changes when the temperature is varied. When the temperature is lower than room temperature, MTPAM JPs with RTP can monitor temperature change between 5 °C and 25 °C and the color of TPAM hemisphere gradually fade from magenta to white. Similarly, the MTPAM JPs with BTP show a sensitive temperature range from 25 °C to 45 °C and the color vary from mazarine to white. By using the mixture of the BTP/RTP as the thermochromic materials, the MTPAM JPs with a wide temperature sensitive range (5–45 °C) are realized. With the increasing of the temperature, its color changes from red to blue and white. As a result, the MTPAM JPs will be favorable in temperature sensor or color display.

Besides the temperature sensitivity, the MTPAM JPs also show high response to the magnetic field. The orientation and motion of MTPAM JPs can be smoothly switched under a rotating magnetic field induced by an external magnet (Fig. 7(l–p)). Here, a MTPAM Janus sphere is put into the culture dish with ethanol solution at room temperature (25 °C). A high-speed camera connected with a microscope lens is used to record the trajectory of this sphere. It is available to manipulate a single Janus spheres by a permanent magnet. When the magnet is applied, the Janus spheres rotate, and the black (Fe_3_O_4_) hemispheres orient towards the permanent magnet. The optical images for the rotational process of a single Janus spheres are taken at regular angle intervals of 45°. Obviously, by rotating the external magnetic field, the direction and motion of the JPs can be easily controlled. Therefore, the MTPAM Janus sphere also possesses high potential in smart magnetic switch.

**Fig. 8:**
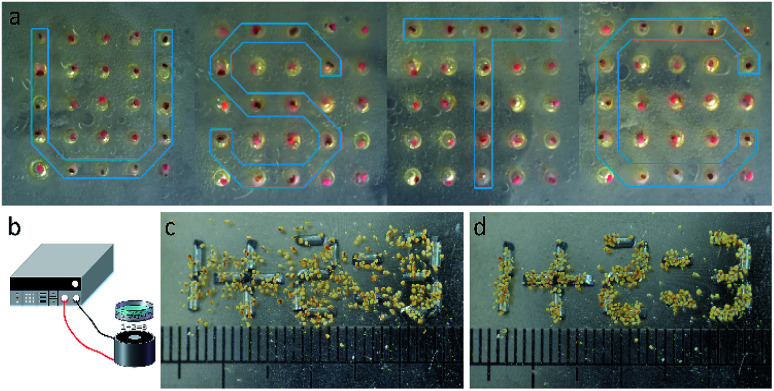
(a) The magneto-thermochromic display prepared by the dual responsive MTPAM JPs. (b) Schematic diagram the experimental device. (c and d) The pattern forming process.

Based on the above discussion, the MTPAM Janus sphere is believed to be widely applied in magnetic manipulation and temperature sensor areas. In this work, a magneto-thermochromic display based on the dual responsive MTPAM JPs is constructed. Firstly, a PDMS board was punched by laser with 25 600 × 600 μm holes (DeLong Laser, UP-D) and each MTPAM Janus sphere was placed in these holes. Because MTPAM JPs can switch under variable magnetic fields, the final platform can be served as the pixel units in a bead display. As shown in Fig. 8a, the display screen could show different arrays *via* a magnet pen based on magnetoresponse at 5 °C. Different from the previous magnetic display, the MTPAM JPs display is facilely accessed to magnetic-field-induced patterns with excellent temperature-dependent color. Therefore, the MTPAM JPs exhibited high potency in magneto-colorful display due to the magnetic rotation and temperature response color and finally the “USTC” pattern was achieved. Additionally, the electromagnet, iron wires segment, a programmable power supply were also used to assemble another display screen (Fig. 8b). In detail, the iron wires were cut into a length of 3 mm, which was used as pixel units to generate a special magnetic field. The length of JPs was 500 μm, and the JPs were assembled above the pixel units. When the current was applied, the display screen was turned on and the JPs would spontaneously aggregate under the magnetic field. Consequently the pattern was formed (Fig. 8d).

## Conclusions

In summary, a simple microfluidic device is developed to construct magneto-thermochromic Janus spheres by integrating Fe_3_O_4_ microspheres, thermochromic particles, and polyacrylamide matrix together. The magnetic field is applied for preparing the Janus spheres because the magnetic Fe_3_O_4_ microspheres can be concentrated to one pole of the sphere during the preparation. This magneto-thermochromic Janus sphere shows a distinct color change over a wide temperature range (5–45 °C). The detailed formation mechanism of the magneto-thermochromic Janus sphere is discussed. Owning to the wonderful magnetic responsibility and temperature dependent color, these JPs show high potential in magnetic detection, temperature sensor, and colorful displays. We believe that more sophisticated and versatile unit approaches and applications in environment monitor and smart display could be further developed.

## Conflicts of interest

There are no conflicts of interest to declare.

## Supplementary Material

RA-009-C9RA02892G-s001
