# Supplementary material for: Magneto-thermochromic coupling Janus sphere for dual response display
Source: RSC Adv. 2019 Jun 7;9(31):17959–66. doi: 10.1039/c9ra02892g (PMC9064662; doi:10.1039/c9ra02892g)
Supplement: RA-009-C9RA02892G-s001 [file RA-009-C9RA02892G-s001.pdf]

Electronic Supplementary Material (ESI) for RSC Advances.
This journal is © The Royal Society of Chemistry 2019

Supporting Information

Magneto-thermochromic Coupling Janus Sphere for Dual Response Display

Yiwen Cui, Yu Wang*, Jie Wu, Xiaokang He, Shouhu Xuan* and Xinglong Gong *

CAS Key Laboratory of Mechanical Behavior and Design of Materials, Department of Modern Mechanics, CAS Center for Excellence in Complex System Mechanics, University of Science and Technology of China, Hefei, Anhui 230027, China.

E-mail: gongxl@ustc.edu.cn (X. Gong); Tel: +86 551 63600419.

E-mail: xuansh@ustc.edu.cn (S.Xuan); Tel: +8655163601702.

E-mail: wyu@ustc.edu.cn(Y. Wang); Tel: +8655163601236.

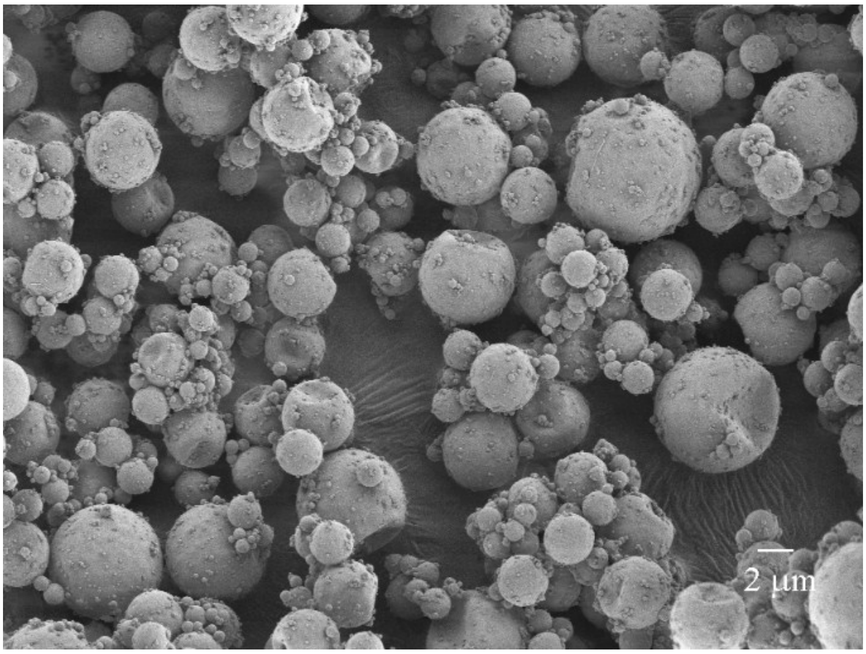

Fig. S1 Scanning electron microscope (SEM) image of blue thermochromic particles.

Table 1: Composition of red thermochromic paticles

CAS	Chemical Name	w/w (%)
192190-91-3	1-[4-[(E)-2-[4-(dibutylamino)phenyl]ethenyl]phenyl]-2,2,2-trifluoroethanone	45%
9002-88-4	poly(ethylene)	5%
52238-92-3	(4E)-4-[[2-chloro-5-(trifluoromethyl)phenyl]hydrazinylidene]-N-[2,5-dichloro-4-[[(4Z)-4-[[2-chloro-5-(trifluoromethyl)phenyl]hydrazinylidene]-3-oxonaphthalene-2-carbonyl]amino]phenyl]-3-oxonaphthalene-2-carboxamide	50%

Table 2: Composition of blue thermochromic paticles

CAS	Chemical Name	w/w (%)
192190-91-3	1-[4-[(E)-2-[4-(dibutylamino)phenyl]ethenyl]phenyl]-2,2,2-trifluoroethanone	45%
9002-88-4	poly(ethylene)	5%
81-77-6	Vat Blue 4	50%
